# Prediction and Decomposition of Efficiency Differences in Chinese Provincial Community Health Services

**DOI:** 10.3390/ijerph15102265

**Published:** 2018-10-16

**Authors:** Qian Liu, Bo Li, Muhammad Mohiuddin

**Affiliations:** 1 Department of Social Medicine and Health Administration, School of Public Health, Tianjin Medical University, Tianjin 300070, China; liuqian2010@tmu.edu.cn; 2 International College of Business and Technology, Tianjin University of Technology, Tianjin 300384, China; great2011818@126.com; 3 School of Business and Economics, Thompson Rivers University, Kamloops, BC V2C 0C8, Canada

**Keywords:** community health service, Super-SBM DEA, efficiency, GM (1,1) grey prediction, Theil index decomposition

## Abstract

The objective of this paper is to analyze the provincial efficiency of the Chinese community health care service and its differences. This study allows us to predict the provincial differences in the efficiency of the Chinese community health care service from 2017 to 2026. This study analyzes the contributions of inter-regional and intra-regional differences in the total efficiency difference. We use the Super-SBM (Slacks-based Model) data envelopment analysis (DEA) model, Grey Model GM (1,1) for grey prediction, and the group-based Theil index decomposition method to study Chinese provincial panel data from 2008 to 2016. Results show that a fluctuating trend existed in the average provincial efficiency of community health services from 2008 to 2016. The community health services in a considerable number of provincial areas were inefficient. This study also reveals that there existed apparent inter-provincial differences in efficiency in Chinese community health services. The inter-provincial differences of the efficiency of Chinese community health services revealed by the Theil index declined at a relatively slow pace. With regard to the provincial efficiency difference of the Chinese community health service, the intra-regional efficiency difference is the most important structural reason for the overall efficiency difference, which explains the overall difference to a large extent. The inter-regional efficiency difference among the eastern, central, and western regions becomes the secondary structural reason, which should not be ignored. In conclusion, focus should be put on restructuring the investments into medical resources for community health service in each Chinese province. More attentions should be put into narrowing the inter-regional efficiency differences of the Chinese provincial community health service. The strategies targeted at reducing the inter-regional efficiency differences should not be ignored, so as to facilitate the improvement of overall efficiency of the Chinese community health service.

## 1. Introduction

The community health care service plays a fundamental role in a country’s public health service system. The development of the community health service system has an apparently positive role in the optimization of the public health service system, realization of fairness, control of the rapid growth in the medical expenses of public health services, and the improvement of the health level of residents.

Inadequate healthcare resource availability and their optimal allocation is the main factor that confronts most of the world’s societies [1]. Although it has been almost a decade since the global financial crisis of 2008 took place, healthcare has continuously been one of the hardest hit sectors following the crisis. The healthcare sector experienced a slowdown in healthcare-related spending around the world. Even rich industrial countries such as Organization of Economic Cooperation and Development (OECD) members are also undergoing the pressure of reduction in healthcare expenditure [2]. This situation calls for a fast transformation from spending-driven to productivity-driven public health services, especially for community healthcare services of all countries.

Facing the ever-growing challenges, it is critical for healthcare institutions to be aware of the importance of improving both the efficiency and the value of investment in the healthcare sector [3,4]. Therefore, it is important to identify efficiency measurement methods that can satisfy our needs. Data Envelopment Analysis (DEA) is one such measurement and benchmarking tool, especially in the healthcare sector [5,6,7,8,9,10,11].

In community health care service research, the issue of efficiency is always the focus of existing literature. Despite the continuing growth of Chinese medical and health care investments, the demand for public health services for both urban and rural residents is increasing at an ever-faster pace. Therefore, the problems of the relative scarcity of health care service resources need to be addressed. It is of great significance to study how to use relatively limited community health service resources in a more efficient way, to meet the growing demand of health care services.

Furthermore, China has a vast territory. There exist obvious differences in community health service resources among provinces and regions. Apparently, the differences will definitely be reflected in the efficiency of the community health service, which, if left uncontrolled would affect the improvement of overall efficiency of Chinese community service. Therefore, it is of great importance to measure the provincial difference of the Chinese community health service efficiency, and to explore effective ways to narrow the differences.

Based on the domestic and foreign existing literature, the data envelopment analysis method is frequently used to study the efficiency of community health care services [12,13,14,15]. Some studies have focused on the issue of community health service resource allocation [16,17]. For the measurement of differences of health resource allocation, inequality measures such as the Gini coefficient are often used in current literature [18,19,20]. Therefore, based on Chinese provincial panel data from 2008 to 2016, this paper uses the DEA method to measure the efficiency of community health services, and uses the Theil index to measure the provincial differences of community health service efficiency. Furthermore, the GM (1,1) grey prediction model is adopted to predict the changing trends of Chinese community health service efficiency differences from 2017 to 2026, and the group-based Theil decomposition method is applied to analyze the sources of efficiency difference, so as to provide political suggestions for the improvement of the overall efficiency of the Chinese community health service.

## 2. Method and Data

### 2.1. Super-SBM DEA Model

There are two approaches in efficiency analysis; parametric and nonparametric ones [21]. The stochastic frontier analysis (SFA) is a typical parametric approach, and data envelopment analysis (DEA) is a typical nonparametric approach. Compared with SFA, DEA does not require relative price information and a specific functional form for a production possibility frontier. The DEA method was first put forward by Charnes et al. [22]. The DEA approach has already been widely applied for evaluating efficiencies in health care systems [23,24,25]. The application of DEA methods are widely accepted due to advantages such as simultaneous use of multiple inputs and outputs variables, and no requirement of a mathematical specification of the production function [26,27,28,29,30].

The radial scheme and the non-radial scheme are two typical schemes in DEA. The slacks-based measure (SBM) model is a representative non-radial scheme model. Compared with the radial scheme model, the SBM model does not adopt the assumption of proportional change in input or output resources, but aims to obtain maximum rates of change of inputs and/or outputs [31,32]. Therefore, the SBM model could overcome the traditional decision-misleading problem of using an efficiency score as the only index in the evaluation of decision making units (DMUs) by eliminating non-radial slacks [31,33]. Compared with the traditional DEA model, the super efficiency SBM model can not only deal with the undesirable outputs, but also can further compare the effective DMUs [34].

The SBM was introduced by Tone [31]. There are *j* = 1, …, n, DMUs with *X_ij_* representing multiple inputs (*i* = 1, …, m) and *Y_rj_* (*r* = 1, …, s) representing multiple outputs of DMU_j_. While *s_i_*^−^ and *s_i_*^+^ are the slack variables for the *i*th input and the *r*th output, *λ_j_* represents the weight of DMU_j_ during the performance measurement of DMU_0_. Under the SBM model will be as follows:

(1) $$\min\rho =\frac{1-\frac{1}{m}\sum_{i=1}^{m}s_{i}^{-}/x_{i0}}{1+\frac{1}{s}\sum_{r=1}^{s}s_{r}^{+}/y_{r0}}$$

s.t. $x_{0}=X\lambda +s^{-},\;y_{0}=Y\lambda +s^{+},\lambda \geq 0,s^{-}\geq 0,\;s^{+}\geq 0$.

If the optimal solution for SBM is (ρ*, λ*, s^−^* and s^+^*), then DMU_0_(*x*_0_,*y*_0_) is SBM-efficient (efficient status) if ρ* = 1, which corresponds with s^−^* = 0 and s^+^* = 0. If ρ* < 1, DMU is inefficient (inefficient status).

In most efficiency evaluation research, there exists a common phenomenon such that plural DMUs have an “efficient status” denoted by 100% (ρ* = 1), so it is important to discriminate between these efficient DMUs for efficiency ranking and influencing factors [35]. To improve and assume that DMU_0_ is SBM efficient (ρ* = 1), Tone [36] proposed the slack-based super-efficiency DEA model, which can be referred to as the Super-SBM DEA model. The model can provide better precision and accuracy in the calculation of the DEA scores. Under a variable return-to-scale (VRS) assumption, the model can be expressed as follows:

(2) $$\min\delta =\frac{\frac{1}{m}\sum_{i=1}^{m}\bar{x_{i}}/x_{i0}}{\frac{1}{s}\sum_{r=1}^{s}\bar{y_{r}}/y_{r0}}$$

$$s.t.\;\;\bar{x}\geq \sum_{j=1,\neq 0}^{n}\lambda_{j}x_{j},\;\bar{y}\leq \sum_{j=1,\neq 0}^{n}\lambda_{j}y_{j},\bar{x}\geq x_{0},\;\mathrm{and}\;\bar{y}\leq y_{0},\;\sum_{j=1,\neq 0}^{n}\lambda_{j}=1,\;\bar{y}\geq 0,\;\lambda \geq 0.$$

where *s* denotes input and output slacks, *λ* denotes weight, *x* denotes input, and *y* denotes output. The target function value of *δ*, which is the Super-SBM efficiency value of the DMU, can be more than 1.

### 2.2. GM (1,1) Grey Prediction Model

The Grey System theory was first proposed by Deng [37] in 1982. Since then, it finds wide-ranging applications in various research fields [38,39]. GM (1,1) is a well-studied prediction model based on the Grey System theory, which is known as a first-order model with one variable [40]. Compared with the traditional statistical forecast models, the historical data requirement for model construction of grey predictions is very flexible, which can be minimized to four sample points. Furthermore, the GM (1,1) model not only has a simple principle, less samples, easy calculation, high forecasting accuracy, and easy inspection, but it can also preprocess the original data, obtain better smoothness, and predict more effectively [41]. Therefore, the Grey GM (1,1) model has been widely applied in many fields such as economics, science, and education [42].

According to GM (1,1), using first-level cumulative generating operation on a raw data sequence (*X*^(0)^), the 1-AGO (Accumulation Generating Operation) sequence (*X*^(1)^) could be obtained. Through mean generation with consecutive neighbors based on *X*^(1)^, *Z*^(1)^ sequence could be obtained. Then, the basic form of the GM (1,1) model, a linear first-order differential equation of the estimation of the cumulative sequence, could be established. By solving the equation, the corresponding predictive values could be obtained through least square method. The detailed formula of GM (1,1) are seen as below:

- Step 1: Given an original sequence as follows:
  (3) $$x^{\left(0\right)}=\left\{x^{\left(0\right)}\left(1\right),\;x^{\left(0\right)}\left(2\right),\;x^{\left(0\right)}\left(3\right),\ldots ,x^{\left(0\right)}\left(i\right),\ldots ,x^{\left(0\right)}\left(n\right)\right\}$$
  where, $x^{\left(0\right)}\left(i\right)$ is the value for the time period *i* (*i* = 1, 2, …, *n*).
- Step 2: A new sequence *x*^(1^^)^ can be generated by a one-time accumulated generating operation 1-AGO) based on the original sequence *x*^(^^0^^)^, which is:
  (4) $$x^{\left(1\right)}=\left\{x^{\left(1\right)}\left(1\right),x^{\left(1\right)}\left(2\right),x^{\left(1\right)}\left(3\right),\ldots x^{\left(1\right)}\left(i\right),\ldots ,x^{\left(1\right)}\left(n\right)\right\}$$
  where, $x^{\left(1\right)}\left(i\right)=\sum_{j=1}^{i}x^{\left(0\right)}\left(j\right)$.
- Step 3: A first-order differential equation with one variable is expressed as follows:
  (5) $$\frac{dx^{\left(1\right)}}{dt}+ax^{\left(1\right)}=b$$
  where, *a*, *b* are the developing coefficient and the grey input coefficient, respectively. These two coefficients can be determined by the least squares method as follows:
  (6) $${\left[a,b\right]}^{T}={\left(P^{T}P\right)}^{-1}P^{T}Q$$
  where:
  $$P=\left[\begin{array}{c} \begin{array}{c} -\left(x^{\left(1\right)}\left(1\right)+x^{\left(1\right)}\left(2\right)\right)/2\;\;\;\;\;\;\;\;\;\;\;\;\;\;\;\;\;1\\ -\left(x^{\left(1\right)}\left(2\right)+x^{\left(1\right)}\left(3\right)\right)/2\;\;\;\;\;\;\;\;\;\;\;\;\;\;\;\;\;1\\ \ldots \end{array}\\ -\left(x^{\left(1\right)}\left(n-1\right)+x^{\left(1\right)}\left(n\right)\right)/2\;\;\;\;\;\;\;\;\;1 \end{array}\right]$$
  and:
  $$Q={\left[x^{\left(0\right)}\left(2\right),x^{\left(0\right)}\left(3\right),\ldots ,x^{\left(0\right)}\left(n\right)\;\right]}^{T}$$

Solving Equation (6), the time response function of the GM (1,1) is given by:

(7) $${\hat{x}}^{\left(1\right)}\left(i\right)=\left[x^{\left(0\right)}\left(1\right)-\frac{b}{a}\right]e^{-\mu \left(i-1\right)}+\frac{b}{a}\;\;\;\;\;\;\left(i=2,3,\ldots ,n\right)$$

The forecasted series ${\hat{x}}^{\left(0\right)}$ can be calculated as follows:

(8) $${\hat{x}}^{\left(0\right)}\left(i\right)={\hat{x}}^{\left(1\right)}\left(i\right)-{\hat{x}}^{\left(1\right)}\left(i-1\right)\;\;\;\;\;\;\left(i=2,3,\ldots ,n\right)$$

where ${\hat{x}}^{\left(0\right)}\left(1\right)={\hat{x}}^{\left(1\right)}\left(1\right).$

### 2.3. Theil Index Decomposition Method

The Theil index calculation method is used as an index to measure the provincial or provincial differences. The Theil index was first proposed by Theil [43] to measure differences between samples. In recent years, it has been widely used to measure regional inequalities in income and energy intensity [44,45]. The advantage of the Theil index is that it can measure the contribution of intra-group and inter-group gaps to total gaps, thus avoiding the calculation of absolute values.

Supposing N (*i* = 1, 2, …, N) geographical areas are under consideration, a commonly used inequality measure is the generalized entropy indices, which can be formulated as follows:

(9) $$GE\left(\beta \right)=\frac{1}{\beta \left(\beta -1\right)}\underset{i}{\sum }p_{i}\left[{\left(\frac{Eff_{\mu }}{Eff_{i}}\right)}^{\beta }-1\right]$$

where *Eff* denotes the community health service efficiency of each geographical area (e.g., provincial area), *β* is a parameter that reflects the sensitivity of the inequality measure to the distributional changes in the aggregate indicator (i.e., *Eff* here), the subscript *μ* denotes the average of the all geographical areas, and *p* is the weight of each area. The selection of the parameter *β* depends on the purpose of a study. When *β* = 0 or *β* = 1, the generalized entropy measure is also known as the Theil index.

Based on the calculation result of the Theil index, the Theil index decomposition method is used to analyze sources of difference, which could attribute the overall difference into an intra-provincial difference and an inter-provincial difference. The specific decomposition method is shown below:

(10) $$Theil=\;Theil_{W}+Theil_{B}$$

(11) $$Theil_{W}=\overset{m}{\underset{p=1}{\sum }}\left(\frac{n_{p}}{n}\frac{{\bar{e}}_{p}}{\bar{e}}\right)Theil_{p}$$

(12) $$Theil_{B}=\overset{m}{\underset{p=1}{\sum }}\frac{n_{p}}{n}\left(\frac{{\bar{e}}_{p}}{\bar{e}}\right)\ln\left(\frac{{\bar{e}}_{p}}{\bar{e}}\right)$$

where, *m* represents the number of regional groups, *n_p_/n* represents the proportion of the number of provinces of every region, *ē_p_/ē* represents the proportion of the index value of each region, and *Theil_W_* and *Theil_B_* represent the intra-regional Theil index value and inter-regional Theil index value respectively.

### 2.4. Data

In order to measure the community health service efficiency in China, this paper used 30 provinces, autonomous regions, and municipalities directly under the central government as the decision-making unit. Due to data accessibility and availability issues, the data of Tibet was not included in the analysis. The number of inputs/outputs to include in a DEA model is a key choice to make. The number of observations should be at least as large as four times the numbers of inputs and outputs. Therefore, based on the representativeness of the indicators, combined with the common practice of existing research, three inputs and three outputs were included as follows:

(1) Input indicators included: the number of community health service centers (stations), the number of community health care technical workers, and the number of community health service beds in each region.
(2) Output indicators include: the number of community health service diagnosed and treated visits, the number of community health services inpatients, as well as the bed utilization ratio of community health services in each area.

In view of the study purpose and data integrity, this paper selected Chinese provincial panel data from 2008 to 2016. The data was extracted from the “Chinese Health Statistics Yearbook” and the “Chinese statistical yearbook of health and family planning”. All the calculations were realized using Microsoft Excel 2010.

## 3. Results and Discussion

### 3.1. Measurement of Chinese Provincial Community Health Service Efficiency

According to the selected input and output variables and data, this paper calculated the efficiency based on the Super-SBM DEA model. The results are shown in Table 1.

According to the results in Table 1, on the one hand, there was a fluctuating trend in the average provincial efficiency of community health services from 2008 to 2016. In 2008, the average community health service efficiency value was 0.834; then it decreased to 0.655 in 2011. After fluctuations from the beginning of 2012, the efficiency reached 0.685 in 2016. On the other hand, from provincial perspective, the community health services in a large number of provinces were inefficient. In 2008, the community health services of only 12 provinces were efficient, including Jilin, Beijing, Shanghai, Jiangsu, Zhejiang, Fujian, Guangdong, Hainan, Chongqing, Sichuan, Qinghai and Ningxia. In 2016, the number of efficient provinces further declined to 10 provinces, including Hebei, Shanghai, Jiangsu, Anhui, Shandong, Hubei, Guangdong, Hainan, Chongqing, and Ningxia. On the whole, from 2008 to 2016, community health services in only six provinces were consistently efficient, including Shanghai, Jiangsu, Guangdong, Hainan, Chongqing, and Ningxia. To sum up, the community health services in a considerable number of provinces were inefficient, and there were apparently differences in the levels of efficiency across the province.

### 3.2. Measurement and Prediction of Efficiency Differences of Chinese Provincial Health Care Services

#### 3.2.1. Measurement of Efficiency Differences of Chinese Provincial Health Service

**Figure 1:**
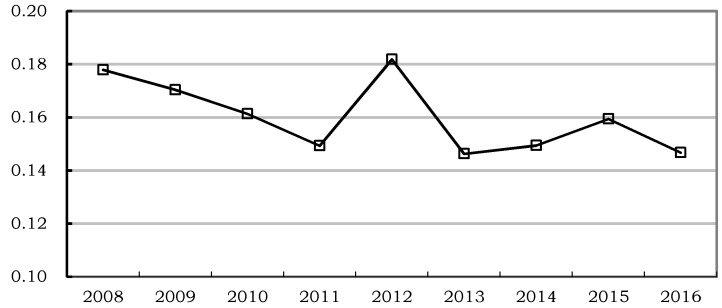
Theil index of the Chinese provincial community health service efficiency.

According to the efficiency measurement of community health service of each province, there existed a significant difference, across the provinces, in efficiency level from 2008 to 2016. Therefore, in order to further quantify the differences, the Theil index (Theil) is adopted to measure the provincial community health service efficiency difference. The results are shown in Figure 1.

Figure 1 displays the provincial efficiency difference of the community health services between 2008 and 2016 in 30 provinces in China. As shown in Figure 1, the Theil index of community health service efficiency in Chinese provinces was 0.1779 in 2008. With the passage of time, the provincial differences of the community health service efficiency declined to 0.1493 in 2011. The Theil index then increased sharply to 0.1818 in 2012, followed by a decline to 0.1468 in 2016. In order to strengthen the community health service institutions, the Ministry of Health of the People’s Republic of China launched a nationwide campaign called “Building up Demonstration Community Health Service Centers” in 2011. Due to the uneven development level of different regions, the response speed and input intensity were also different, which led to different speeds of improvement of community health services in various places. The effect of the campaign was gradually revealed from 2012, which led to a large degree of efficiency gap. As time proceeded, the areas with relatively backward efficiency gradually caught up, so that the gap narrowed. Overall, the provincial differences in the efficiency of Chinese community health services from 2008 to 2016 decreased at a relatively slow pace, which was obviously conducive to the improvement of overall efficiency of community health services in China.

#### 3.2.2. Prediction of Efficiency Difference of Chinese Provincial Health Service

**Figure 2:**
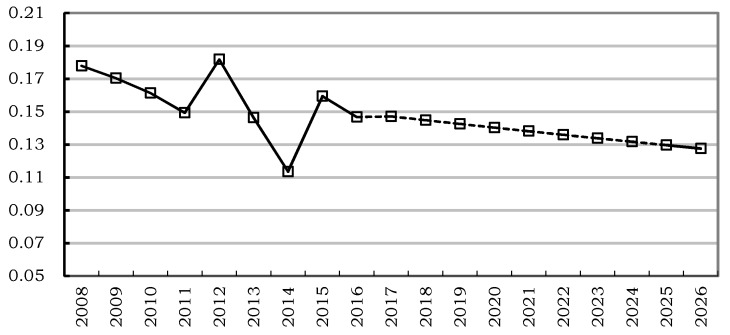
The actual and predicted Theil index of Chinese provincial community health service efficiency.

Based on the measurement of the Theil index for the provincial efficiency differences of community health services from 2008 to 2016, the GM (1,1) grey prediction model can be established. Through calculation, model parameters could be obtained, and the reliability of the model could be tested, where *a* = 0.0158, *b* = 0.1711, and the average relative prediction error is 5.30%. According to the relevant research, if the average relative error was less than 20%, then the prediction result could be considered as acceptable [19]. Therefore, the prediction result of this study was acceptable. According to the model, the changing trend of the Theil index of Chinese community health service efficiency from 2017 to 2026 could be obtained. The results are shown in Table 2 and Figure 2.

As shown in Table 2 and Figure 2, the Theil index of the provincial differences of Chinese community health service efficiency will be decreased to 0.1276 in 2026, a decline of 13.08% compared to 0.1468 in 2016. The prediction indicates that there will be an overall downward trend in the future for the efficiency difference, but the decline will be taking place at a relatively slow pace, which will definitely hamper the improvement of the overall level of community health service efficiency. Therefore, it is necessary to explore the sources of the provincial efficiency differences in Chinese community health services.

### 3.3. Decomposition of the Efficiency Differences in Chinese Provincial Health Services

**Figure 3:**
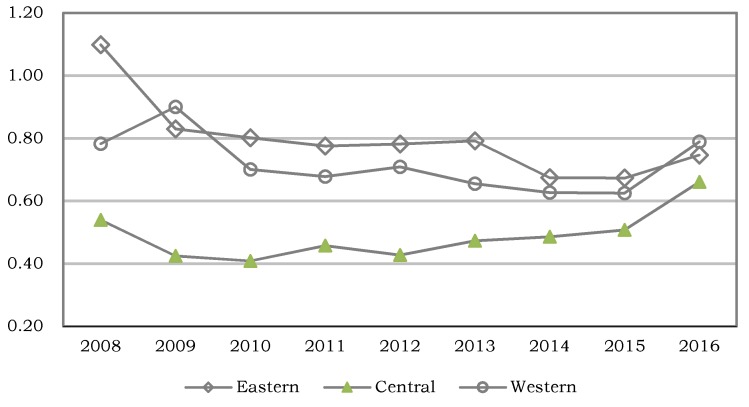
Average values of provincial efficiencies within each region (2008–2016).

Further investigation of the regional efficiency distribution was based on a three-region division of the provinces proposed in “China Health and family planning Statistical Yearbook”, in which a total of 11 provincial areas were included in the eastern region, including Beijing, Tianjin, Hebei, Liaoning, Shanghai, Jiangsu, Zhejiang, Fujian, Shandong, Guangdong, Hainan; a total of eight provincial areas were included in the central region, including Shanxi, Jilin, Heilongjiang, Anhui, Jiangxi, Henan, Hubei, and Hunan; a total of 11 provincial areas were included in the western region, including Inner Mongolia, Guangxi, Chongqing, Sichuan, Guizhou, Yunnan, Shaanxi, Gansu, Qinghai, Ningxia, and Xinjiang. According to the above regional division method, this paper further calculated the average value of the provincial efficiencies within each region from 2008 to 2016. The results are shown in Table 3 and Figure 3.

As shown in Table 3, on average, from 2008 and 2016, the average values of the provincial efficiencies within each region from high to low were as follows: eastern (0.797), western (0.718), and central (0.487). The result reveals that there existed significant regional differences in the provincial efficiency of the Chinese community health service. As clearly shown in Figure 3, the average provincial efficiency within the eastern region was slightly higher than that within the western region, except for 2009 and 2016, while the average provincial efficiency within the central region was significantly lower than those within the eastern and western regions. This situation obviously hindered the overall improvement of Chinese community health service efficiency.

There are various explanations for the above results. The first reason lies in the difference of medical and health conditions. The medical infrastructure in the eastern region was generally considered to be relatively developed and complete, so that people’s medical needs could be effectively and timely met. The second reason comes from population density. The accessibility of medical services formed by the population density of the central and western regions and the eastern region will also increase its efficiency. The third reason could be attributed to economic development. The economies in the central and western regions are relatively backward, unable to guarantee adequate public finances for the medical and health departments, and individuals are not able to purchase medical and health services. In addition, there is a shortage of medical personnel and equipment in the region, especially in comparison with the population density. The socio-economic backwardness also hinders the improvement of health care service levels to a certain extent.

Another issue that needs to be pointed out is that as empirical research shows, the average provincial efficiency of the Chinese community health services in western regions is apparently higher than the average efficiency in the central region, and even higher than that of the eastern region in 2016. The results show that the western region has created more output by using relatively less medical and health care resource inputs at community level.

Theoretically speaking, the higher the proportion of more advanced community health centers and institutions, the higher the efficiency of health care services in that province. More advanced centers and institutions mean a higher level of medical technology, practices, and operation management, which leads to more effective medical resources utilization. However, in reality, often the case is that in economically developed eastern and central regions, patients have a higher purchasing power of health care services, and a higher sensitivity to medical care service, which usually leads to the phenomenon of “over-treatment” or “excess treatment”. Through over-treatment or excess treatment, excessive consumption of relatively scarce medical resources is directed to many common diseases, which causes a supply shortage and underserved usage of community health and medical care resources. This phenomenon is especially apparent in the “first-tier cities” such as Beijing, Shanghai, etc. or in relatively more developed areas that are often located in the eastern or central regions, preventing further improvement of efficiency in health services and medical institutions in these regions.

Therefore, it is necessary to use a relatively objective method to accurately measure the sources of the overall efficiency differences. The use of an effective method can provide reasonable theoretical basis for finding effective ways to narrow the overall efficiency differences in the Chinese community health service. Under the above consideration, this paper uses a group-based Theil index decomposition method to investigate the contributions of inter-regional differences and intra-regional differences, so as to find the sources of the overall efficiency difference. The results are shown in Table 4.

(1) In the provincial efficiency differences of Chinese community health services, the intra-regional efficiency differences become the most important source for overall efficiency differences, and inter-regional efficiency differences among the eastern, central, and western region becomes the secondary source. In 2008, the contribution of intra-regional efficiency differences to the overall difference was 79.5%, and the contribution of inter-regional efficiency differences to the overall difference was 20.5%. Then, the contribution of intra-regional efficiency differences increased with fluctuations. In 2016, the contribution of intra-regional efficiency differences increased to 98.4%, and the contribution of inter-regional efficiency differences declined to only 1.6%.
(2) The changing trends of contributions of intra-regional efficiency differences vary tremendously. The contribution of intra-regional efficiency differences of the eastern region declined from 51.6% in 2008 to 39.3% in 2016, which was the highest among all the regions, except for 2012. The contribution of the intra-regional efficiency differences of the western region increased from 22.6% in 2008 to 36.7% in 2016, which was the second highest among the three regions. The contribution of the intra-regional efficiency differences of the central region increased from 5.3% in 2008 to 22.4% in 2016, which was the lowest among the regions.

The decomposition result revealed an important changing trend: in explaining the sources of overall efficiency differences in Chinese community health service, the traditional eastern–central–western division paradigm is gradually losing its explanatory power. With fast-growing urbanization in China, more and more people are moving into densely populated urban areas in all of the regions, which lead to economies of scale and economies of scope for community health services, and helps the shrinkage of regional difference of efficiencies.

Another noteworthy changing trend is that although intra-regional efficiency differences in the eastern region are reducing, intra-regional efficiencies in both the central and western regions are changing dramatically. One possible reason may come from institutional sources. The social security system and the medical insurance system of the provinces in the eastern region are relatively mature, which can ensure the effective coverage of medical services as public goods. In contrast, in the provinces in central and western regions, the gap is becoming larger, due to difference in public input for building up a social security network.

The second possible reason comes from economic development. The established relationship between regional economic development, and medical and health efficiency, is that the higher the degree of economic development in a region, the more likely it is able to provide financial support to improve the public health service system and technical conditions, and obtain a higher level of efficiency. The disparity in economic development of provinces in the central or western regions is relatively large, making it difficult to maintain a balanced standard for community health services in all provinces of a certain region. Hence, the intra-regional efficiency difference is increasing in the central and western regions.

Differences in educational level might be another reason. The gap of individual educational level and health knowledge among provinces in each region are negligible. Generally, a higher education level makes the residents more capable of improving their ability to supervise community health institutions, promoting the efficiency of community health services. In each region, there are provinces that enjoy higher individual educational levels, and there, are of course, a certain number of provinces that have to deal with the problem of relatively lower individual educational levels, which in turn impacts the intra-regional efficiency difference in community health services, especially in the central and western regions.

## 4. Conclusions

This paper uses the Chinese provincial panel data of 30 provinces from 2008 to 2016, and the data envelopment analysis (DEA) model, to measure the efficiencies of Chinese provincial community health services. Then, we calculate the Theil index to measure the provincial differences of efficiency in community health services, and predict the provincial differences in the efficiency of the Chinese community health services from 2017 to 2026 using a GM (1,1) grey prediction model. Based on the results of calculation and prediction, this paper further uses the group-based Theil index decomposition method to study the sources of overall provincial efficiency differences of the Chinese community health service.

The results of this study show that:

(1) There was a fluctuating trend in the average provincial efficiency of the community health service from 2008 to 2016. The community health services in a considerable number of provinces were inefficient.
(2) There were apparent provincial differences in the efficiency of Chinese community health services. The provincial differences of efficiency of Chinese community health services from 2016 to 2008 reflected by the Theil index declined at a relatively slow pace with fluctuations. Overall, although the regional gap of efficiency of the Chinese community health service declined in recent years, the gap is still apparent and it needs to be narrowed.
(3) In the provincial efficiency differences of Chinese community health services, the intra-regional efficiency difference is the most important source for the overall efficiency difference, which explains the overall difference to a large extent. The inter-regional efficiency difference among the eastern, central, and western regions was the secondary source, although it should not be ignored.

The results of the study can provide a reference for political decision-making in reducing the provincial differences, and for improving the overall efficiency of the Chinese community health service.

First of all, focus should be put on restructuring investments into medical resources for community health services in each Chinese province. The restructuring efforts can improve the medical resource allocation and reconfiguration capacity, in order to narrow down the provincial efficiency difference and to enhance the overall efficiency in the Chinese community health services. Therefore, with continual financial support and investment in the community health services, it is also necessary to strengthen the mechanisms and practices of supervision of public resource spending. Mechanisms need to be implemented to prevent inefficient use of public resources by “over-treatment” or “excess-treatment”. Policy makers need to focus on optimizing resource allocation and ensuring the sustainable improvement of the efficiency of community health services.

Secondly, more attention should be put onto narrowing the inter-regional efficiency difference of Chinese provincial community health services. Therefore, beneficial regional policies should be formulated to encourage effective coordination and allocation of intra-regional community health service resources. Furthermore, an evaluation mechanism using efficiency as the indicator should be built up and strengthened, to help narrow intra-regional efficiency differences. For each region, especially for eastern regions, community health service system development strategies should be made according to regional characteristics, so as to narrow the intra-regional efficiency differences through inter-provincial assistance.

Finally, a strategy targeted at reducing the inter-regional efficiency differences should not be ignored. Therefore, effective inter-regional community health service development strategies should be made based on specific conditions and overall planning, so as to activate positive regional interactions to improve the overall efficiency of Chinese community health services. Focusing on the steady improvement of the efficiency of the eastern region, effective measures should be adopted to promote trans-regional flows of community health service resources, and to strengthen support and supervision for relatively backward regions, so as to facilitate the improvement of the overall efficiency of the Chinese community health service.

## Figures and Tables

**Table 1:** Results of Chinese provincial community health service efficiency (2008–2016).

No.	Prov.	2008	2009	2010	2011	2012	2013	2014	2015	2016	Avg
1	Beijing	1.053	0.243	0.384	0.377	0.368	0.366	0.295	0.164	0.252	0.389
2	Tianjin	0.253	0.196	0.262	0.350	1.013	1.009	1.000	0.576	0.235	0.544
3	Hebei	0.448	0.384	0.413	0.368	0.328	0.344	0.313	0.304	1.057	0.440
4	Shanxi	0.409	0.309	0.322	0.284	0.287	0.286	0.260	0.218	0.461	0.315
5	Inner Mongolia	0.331	0.294	0.247	0.258	0.236	0.244	0.225	0.228	0.453	0.280
6	Liaoning	0.483	0.390	0.372	0.342	0.329	0.413	0.421	0.336	0.492	0.398
7	Jilin	1.034	0.187	0.203	0.277	0.265	0.231	0.260	0.241	0.193	0.321
8	Heilongjiang	0.387	0.234	0.280	0.274	0.213	0.235	0.273	0.305	0.241	0.271
9	Shanghai	1.889	1.510	1.412	1.376	1.278	1.268	1.000	1.339	1.060	1.348
10	Jiangsu	1.091	1.093	1.170	1.118	1.102	1.106	1.000	1.118	1.083	1.098
11	Zhejiang	1.129	1.002	0.483	0.503	0.495	0.407	0.413	0.391	0.179	0.556
12	Anhui	0.536	0.495	0.369	0.333	0.339	0.401	0.414	0.381	1.063	0.481
13	Fujian	1.421	1.158	1.063	0.750	0.622	0.588	0.575	0.466	0.525	0.797
14	Jiangxi	0.458	0.456	0.341	0.354	0.318	0.410	0.388	0.415	0.574	0.413
15	Shandong	0.435	0.461	0.386	0.534	0.433	0.422	0.397	0.383	1.011	0.496
16	Henan	0.466	0.457	0.469	0.454	0.424	0.450	0.480	0.471	0.808	0.498
17	Hubei	0.551	0.727	0.776	1.067	1.057	1.043	1.000	1.023	1.156	0.933
18	Hunan	0.477	0.532	0.507	0.619	0.513	0.726	0.810	1.004	0.792	0.664
19	Guangdong	1.196	1.334	1.425	1.334	1.278	1.285	1.000	1.095	1.232	1.242
20	Guangxi	0.322	1.077	0.655	0.572	0.560	0.609	0.716	0.584	0.568	0.629
21	Hainan	2.683	1.355	1.451	1.478	1.352	1.501	1.000	1.231	1.075	1.458
22	Chongqing	1.155	1.042	1.100	1.104	1.159	1.168	1.000	1.161	1.194	1.120
23	Sichuan	1.211	1.129	1.114	1.043	1.022	0.842	0.696	0.648	0.716	0.936
24	Guizhou	0.788	1.038	1.037	1.039	0.640	0.671	0.550	0.540	0.736	0.782
25	Yunnan	0.704	1.025	0.719	0.616	0.599	0.560	0.547	0.646	0.687	0.678
26	Shaanxi	0.338	0.394	0.327	0.350	0.386	0.381	0.376	0.324	0.467	0.371
27	Gansu	0.531	0.468	0.382	0.398	0.315	0.348	0.379	0.361	0.573	0.417
28	Qinghai	1.190	1.134	0.625	0.647	0.542	1.000	1.000	1.029	0.602	0.863
29	Ningxia	1.363	1.726	1.000	1.000	1.911	1.000	1.000	1.000	2.147	1.350
30	Xinjiang	0.673	0.574	0.502	0.429	0.424	0.379	0.402	0.351	0.533	0.474
-	Avg	0.834	0.747	0.660	0.655	0.660	0.656	0.606	0.611	0.739	0.685

**Table 2:** The predicted Theil index of Chinese provincial community health service efficiency (2017–2026).

Year	Predicted Theil	Year	Predicted Theil
2017	0.1471	2022	0.1359
2018	0.1448	2023	0.1338
2019	0.1426	2024	0.1317
2020	0.1403	2025	0.1296
2021	0.1381	2026	0.1276

**Table 3:** Average values of provincial efficiencies within each region (2008–2016).

Region	2008	2009	2010	2011	2012	2013	2014	2015	2016	Average
Eastern	1.098	0.830	0.802	0.775	0.782	0.792	0.674	0.673	0.746	0.797
Central	0.540	0.425	0.408	0.458	0.427	0.473	0.486	0.507	0.661	0.487
Western	0.782	0.900	0.701	0.678	0.709	0.655	0.626	0.625	0.789	0.718

**Table 4:** Theil index decomposition results (2008–2016).

Year	Intra-Regional				Inter-Regional	Total	Theil Index
	Eastern	Central	Western	Sum			
2008	51.6%	5.3%	22.6%	79.5%	20.5%	100.0%	0.1779
2009	42.8%	6.9%	27.5%	77.2%	22.8%	100.0%	0.1704
2010	48.8%	7.8%	24.2%	80.7%	19.3%	100.0%	0.1614
2011	45.3%	16.1%	25.4%	86.9%	13.1%	100.0%	0.1493
2012	32.4%	13.6%	39.8%	85.8%	14.2%	100.0%	0.1818
2013	43.2%	18.0%	25.9%	87.1%	12.9%	100.0%	0.1463
2014	46.7%	18.3%	24.4%	89.4%	10.6%	100.0%	0.1494
2015	46.8%	22.5%	26.9%	96.2%	3.8%	100.0%	0.1594
2016	39.3%	22.4%	36.7%	98.4%	1.6%	100.0%	0.1468

## Data Availability

All relevant data are within the paper and its Supporting Information files.
